# Citrate targets FBPase and constitutes an emerging novel approach for cancer therapy

**DOI:** 10.1186/s12935-018-0676-y

**Published:** 2018-11-08

**Authors:** Philippe Icard, Ludovic Fournel, Antoine Coquerel, Joseph Gligorov, Marco Alifano, Hubert Lincet

**Affiliations:** 1 Thoracic Surgery Department, Cochin Hospital, Normandy University of Caen, Unité Inserm 1199, Paris, France; 2 Thoracic Surgery Department, Cochin Hospital, Paris Descartes University, Unité Inserm 1007, 27 rue du Faubourg Saint Jacques, 75014 Paris, France; 3 Normandy University of Caen, Unité Inserm 1075, Caen, France; 4 Oncology Department, Tenon Hospital, Pierre et Marie Curie University, Paris, France; 5 Faculty of Pharmacy of Lyon, CRCL Lyon, Unité Inserm 1052, CNRS 5286, Lyon, France

**Keywords:** Warburg effect, Cancer metabolism, Citrate, FBPase, Cancer therapy

## Abstract

Gao-Min Liu and Yao-Ming Zhang recently published a review entitled «Targeting FBPase is an emerging novel approach for cancer therapy» (Liu and Zhang in Cancer Cell Int 18:36, 2018). In this paper, the authors highlighted how the down regulation or inactivation of FBPase, a rate limiting enzyme of gluconeogenesis, can promote the Warburg effect and cancer growth. In contrast, activation of this enzyme demonstrates anti-cancer effects and may appear as emerging novel approach for cancer therapy. Among the potential activators of FBP listed by Liu and Zhang, citrate was surprisingly not mentioned although it is an activator of FBPase, also demonstrating various anti-cancer effects in pre-clinical studies. Thus, citrate should be tested as a new therapeutic strategy, in particular in clinical studies.

Drs. Gao-Min Liu and Yao-Ming Zhang must be congratulated for their review «Targeting FBPase is an emerging novel approach for cancer therapy» explaining how the down expression or inactivation of this rate limiting enzyme of gluconeogenesis promotes aerobic glycolysis, cancer cell proliferation and dedifferentiation. Therefore, activation of FBPase may have anti-cancer effects, counteracts the Warburg effect and antagonizes chemoresistance [1]. Among the potential activators of FBP listed by Liu and Zhang, citrate was surprisingly not mentioned, although this molecule plays a central role in the metabolism of cancer cells [2]. Physiologically, when energy stores are elevated, citrate is abundant and the activity of (phosphofructokinase 1) PFK1 is nearly switched off [2]. Citrate is also a potent allosteric inhibitor of PFK2 (also called PFKFB (6-phosphofructo-2-kinase/fructose-2,6-biphosphatase) in non-cancer cells (such as cardiac cells [3]) and ascites cancer cells [4]. It is likely that citrate also indirectly inhibits PFK2 through an increased palmitate synthesis [5].

By regulating key regulatory enzymes located at entrance and/or exit of glycolysis (PFK1, PFK2, PK), TCA cycle (PDH, SDH), gluconeogenesis (F1,6BPase) and fatty acid synthesis (ACC), citrate controls anabolic and catabolic pathways, and allows a close adjustment of metabolic flows to ATP production [2].

In cancer cells, the Warburg effect is associated with a decrease production of citrate by mitochondria [6]. This reprogramming metabolism is promoted by over-expression of HIF-1alpha and Myc, activation of Ras, loss of functional p53, all factors promoting the down-regulation of mitochondria [6]. Such down-regulation leads to a reduced production of citrate, ATP and CO_2_, such condition promoting intracellular alkaline pH, a strong activator of PFK-1 [6]. This rate-limiting enzyme of glycolysis is also controlled by a family of regulatory bifunctional enzymes PFKFB. When the kinase activity is promoted, fructose-2,6-bisphosphate (F2,6BP) increases and allosterically activates PFK-1, directing the carbon flux into glycolysis and sustaining cell survival and proliferation [7].

We showed for the first time that administration of citrate to cancer cells arrests cell growth, induces apoptosis (in particular through extinction of expression of the anti-apoptotic factor Mcl-1) and sensitizes chemoresistant cells to cisplatin [8]. Several authors have confirmed on different cancer cell lines that citrate administration (generally at ≥ 10 mM) inhibits glycolysis and ATP production, promotes apoptosis through activation of caspases, and increases response to Bcl-xL inhibitors (for additional references see [6, 9]). Recently, Ren et al. [9] confirmed that citrate decreases resistance to cisplatin (in particular by reducing the expression of Snail and MUC-1), and showed that it inhibits the proliferative IGF-1R/AKT axis, stimulates the suppressive PTEN-eIF2α pathway, and induces tumor cell differentiation (expression of E-cadherin). Importantly, these authors observed that daily oral administration of citrate at a dose of 8 g/kg/day for 1 month reduces tumor growth of several xenograft tumor models in mice (pancreatic cancer, Ras-driven lung tumor and Her2/Neu mammary cancer), increasing significantly the number of infiltrating T-cells in tumors [9].

All these experimental in vitro and in vivo studies give arguments to consider that high-dose citrate administration inhibits cancer cell development. Nonetheless, the mechanisms underlying this process remain unclear. Beside its allosteric inactivation of PFK 1 and 2, reducing or arresting the glycolytic flux, citrate may also exercise other anti-cancer effects by reducing the levels of fructose-1,6-bisphosphate (F1,6BP) and F2,6BP. Indeed, these molecules couple the glycolytic flux with activation of Ras and cell cycle progression, respectively: (i) cytosolic F1,6BP binds Son of sevenless homolog 1 (*Sos1*), a factor promoting the activation of Ras and its downstream targets MEK and ERK [10]; (ii) F2,6BP represses p27Kip1, a potent inhibitor of cyclins D and E regulating G1/S transition, and cyclin-dependent kinase (Cdk)-1 regulating entrance in mitosis [7].

Knowing that both isoforms of FBP (FBP1 and FBP2) are inhibited by Ca^2+^, very probably high concentration of citrate also inactivates these enzymes by this mechanism because citrate is a well-known chelator of calcium. This action should be particularly interesting for treatment of cancers with poor prognosis (such as gastric cancer, brain metastatic breast cancers) expressing low FBP2, because as explained by Liu and Zhang [1], FBP2 is 1000 times more sensitive to inhibition by Ca^2+^.

More studies are needed to nail down the mechanisms underlying citrate effect in cancer cells proliferation and strengthen the proof of principle.
